# Evaluation of a massive open online course for just-in-time training of healthcare workers

**DOI:** 10.3389/fpubh.2024.1395931

**Published:** 2024-10-01

**Authors:** Matthew Charles Strehlow, Jamie Sewan Johnston, Kelly Zhang Aluri, Charles G. Prober, Peter Corrigan Acker, Avinash S. Patil, Aditya Mahadevan, Swaminatha V. Mahadevan

**Affiliations:** ^1^Department of Emergency Medicine, Stanford University, Palo Alto, CA, United States; ^2^Stanford Center for Health Education, Stanford University, Stanford, CA, United States; ^3^School of Medicine, Stanford University, Stanford, CA, United States; ^4^UC Irvine School of Medicine, Irvine, CA, United States

**Keywords:** pandemic, MOOC, online education, LMICs, healthcare worker training

## Abstract

**Introduction:**

COVID-19 created a global need for healthcare worker (HCW) training. Initially, mass trainings focused on public health workers and physicians working in intensive care units. However, in resource-constrained settings, nurses and general practitioners provide most patient care, typically lacking the training and equipment to manage critically ill patients. We developed a massive open online course (MOOC) for HCWs in resource-constrained settings aimed at training bedside providers caring for COVID-19 patients. We describe the development, implementation and analysis of this MOOC.

**Methods:**

From May through June 2020, the course was developed by a multi-disciplinary team and launched on two online platforms in July. The 4-hour course comprises 6 video-based modules. Student knowledge was assessed using pre- and post-module quizzes and final exam, while demographics and user experience were evaluated by pre- and post-course surveys and learning platform data.

**Results:**

From July 17th to September 24th, 30,859 students enrolled, 18,818 started, and 7,101 completed the course. Most participants worked in healthcare (78%) and resided in lower middle- (38%) or upper middle- (20%) income countries. Learners from upper middle-income and lower middle-income countries had higher completion rates. Knowledge gains were observed from pre-module to post-module quizzes and a final exam. Afterward, participants reported increased self-efficacy regarding course objectives, a 0.63 mean increase on a 4-point scale (95% CI [0.60,0.66]). Most participants (93%) would recommend the course to others.

**Conclusion:**

This article demonstrates the potential of MOOCs to rapidly provide access to emerging medical knowledge during a public health crisis, particularly for HCWs in high- and middle-income countries.

## Introduction

Since the first patients with COVID-19 were reported in Wuhan, China, at the end of 2019, cases have been documented on every continent in the world with current estimates in excess of 766 million cases worldwide (1). While countries spanning the entire income spectrum have been impacted, low- and middle-income countries (LMICs) as well as remote and rural healthcare systems in high-income countries (HICs) have been particularly vulnerable — they are at increased risk of being completely overwhelmed, potentially leading to enormous, yet preventable, loss of life (2). Though many health system elements are required to respond to this health crisis, equipping the world’s 60 million healthcare workers with the appropriate knowledge and skills to care for patients with COVID-19 infections is one critical component. Evidence suggests that the early case fatality rate for infected patients quickly dropped 20%, in part, through increased provider experience and the resulting improvements in their routine care (3). This pandemic has highlighted the need for timely, widespread, and effective healthcare worker training focused on the bedside care of patients during global health crises, both now and into the future.

In early 2020, during the initial stages of the pandemic, a number of educational initiatives were launched at global, national, and local levels. The first open-access COVID-19 educational programs focused on caring for critically ill patients requiring invasive mechanical ventilation or public health interventions (4, 5). These programs mirrored synchronous efforts to strengthen infrastructure, such as increased intensive care unit bed capacity and ventilator availability. While the vast majority of patients suffering from COVID-19 did not require invasive mechanical ventilation, the mortality of those who did require intubation remained stubbornly high (6, 7).

In most settings worldwide, and particularly in resource-limited settings, patient care is primarily provided by nurses and other non-physician providers in conjunction with generalist physicians. These providers typically have minimal training in performing advanced airway interventions or managing complex, critically ill patients (8–10). Consequently, the opportunity for improving patient outcomes in most regions of the world was in strengthening the skills necessary for the identification, evaluation and treatment of COVID-19 patients with mild to moderate illness — where appropriate early intervention could obviate the need for invasive mechanical ventilation (11–13). To date, however, there is a paucity of evidence that exigent inservice training targeting providers in LMICs and rural and remote areas (i.e., resource-limited), particularly in the midst of a global health crisis, can be effective for knowledge uptake or influencing clinical practice (14, 15).

Massive open online courses (MOOCs) may be a valuable method of disseminating optimal patient care recommendations to bedside healthcare workers during healthcare crises. Thus far, the vast majority of MOOCs have been designed for, and utilized by, students in North America and Europe (16, 17). Very little published literature has reported on MOOCs designed for inservice training of healthcare workers in LMICs, despite their significant potential impact in this space (18–21).

In addition to identifying effective methodologies for reaching and training healthcare workers in lower resource settings, presenting credible and evidence-based training materials is also critical. An infodemic — where vast amounts of information and misinformation on a topic are readily available leading to confusion and fallacy — has been a consistent challenge during the COVID-19 pandemic, documented across over 30 countries (22, 23). To combat this, a WHO technical consultation on the COVID-19 infodemic has called for strategic partnerships across all sectors, including social media/technology, academia, and civil society, to serve as trusted information sources (24).

Using a self-directed learning theory, we developed and deployed a free-of-charge MOOC entitled “COVID-19: Training for Healthcare Workers” (16). Self-directed learning theory allows individuals to guide their learning, establish their learning objectives, and manage their time based on their needs while still benefiting from access to carefully curated content (25). Our aim was to evaluate the viability of MOOCs to provide rapid access to emerging medical knowledge in the early stages of the COVID-19 pandemic. We hypothesized that participants would show improvement in knowledge gain and self-efficacy toward relevant course topics centered around diagnostic assessment and treatment of COVID-19.

## Materials and methods

### Educational design and delivery

In May through July of 2020, we developed a massive open online course using an international team of 22 emergency medicine physician educators, 3 medical illustrators, 3 video editors, 2 education technology staff, and 1 project manager. The majority of the development team had prior experience designing and building online educational programs. The framework used to construct the learning objectives was the backward design process: (1) Identify desired results; (2) Determine acceptable evidence; and, (3) Plan learning experiences and instruction (26). Learning objectives were defined by a core physician leadership team. The academic faculty that developed the COVID-19 course learning objectives were all medical school faculty from U.S. academic institutions—many with extensive experience designing medical school courses and developing online curricula for medical education. These objectives focused on the essential knowledge and skills deemed necessary for bedside healthcare workers to recognize and care for suspected and confirmed COVID-19 patients in the early and middle stages of their disease course. The course was broken down into 6 modules comprising 15 topic-focused lecture videos (Box 1).

**BOX 1 COVID-19 course modules.**

*Key features and PPE*

- Recognizing key features
- PPE and scene safety

*Clinical assessment*

- Approach to the sick patient
- Shock evaluation at the bedside
- Assessing the dyspneic patient - clinical

*Diagnostic assessment*

- Assessing the dyspneic patient - diagnostic
- Ultrasound in COVID-19

*Early treatment*

- Treating the mildly dyspneic patient
- Treating the moderately dyspneic patient - part 1
- Treating the moderately dyspneic patient - part 2

*Advanced treatment*

- Treating the severely dyspneic patient - part 1
- Treating the severely dyspneic patient - part 2
- Treating the severely dyspneic patient - part 3

*Invasive mechanical ventilation*

- Ventilator management - part 1
- Ventilator management - part 2

We anticipated that the majority of our target learners — self-directed, currently practicing healthcare workers in LMICs and remote and rural settings — would engage in course content via mobile devices. To optimize uptake by these learners, we aimed to produce brief (≤10 min) videos with minimal amounts of simplified text and universal imagery. We followed design principles developed through prior work aimed at using visual styles that resonate across diverse global audiences. The 15 video-based lectures totaled approximately 3-h in length and were accompanied by lecture handouts for review and reference. The course was subsequently launched on two online platforms, Coursera and EdX, on July 17th, 2020. It was also offered as a course on the free mobile application, Digital Medic.

### Course analysis

From July 17th to September 24th, 2020, we recruited all learners who enrolled in the course on Coursera and EdX to participate in our course evaluation. Written informed consent was obtained digitally through a request on the course platforms. Learners who chose not to participate in the evaluation could still access the same course materials. Consenting participants completed questionnaires before and after the course and their course quiz and exam scores were de-identified and included in the analysis. Ethical approval was obtained by the Institutional Review Board at Stanford University (Protocol 57831).

In the baseline survey, participants reported their age, gender, profession, context of employment, education level, and ethnicity/race. Participants’ country of occupancy was obtained from Coursera and EdX directly. Participant knowledge was assessed using pre- and post-module quizzes and a final exam. Knowledge questions were drafted by the faculty content experts who designed the module materials and reviewed by at least two additional faculty and education and instructional design team members. The percentage of correct answers during a participant’s first attempt was used to determine their score. Students were asked to rate their confidence before and after the course in various domains using a 4-point Likert scale. A 4-point scale was chosen in order to force a specific opinion and eliminate a “neutral” option. Each item was rated as follows: 1 = Strongly disagree, 2 = Somewhat disagree, 3 = Somewhat agree, 4 = Strongly agree.

Data from the pre-course demographic survey were summarized using descriptive statistics. To evaluate predictors of course completion, univariate and multivariable logistic regression was used. Paired t-tests were used to detect differences in participant knowledge and self-efficacy scores before and after the course. To assess changes in knowledge, we compared pre-module versus post-module scores, as well as pre-module versus final exam scores. To examine whether there were differences between learners from different backgrounds, knowledge and self-efficacy scores were stratified by occupation and country income level for additional analysis. Data from the participants’ course satisfaction ratings were summarized using descriptive statistics. All statistical analyses were performed using STATA SE version 16.

## Results

### Course engagement

**Figure 1:**
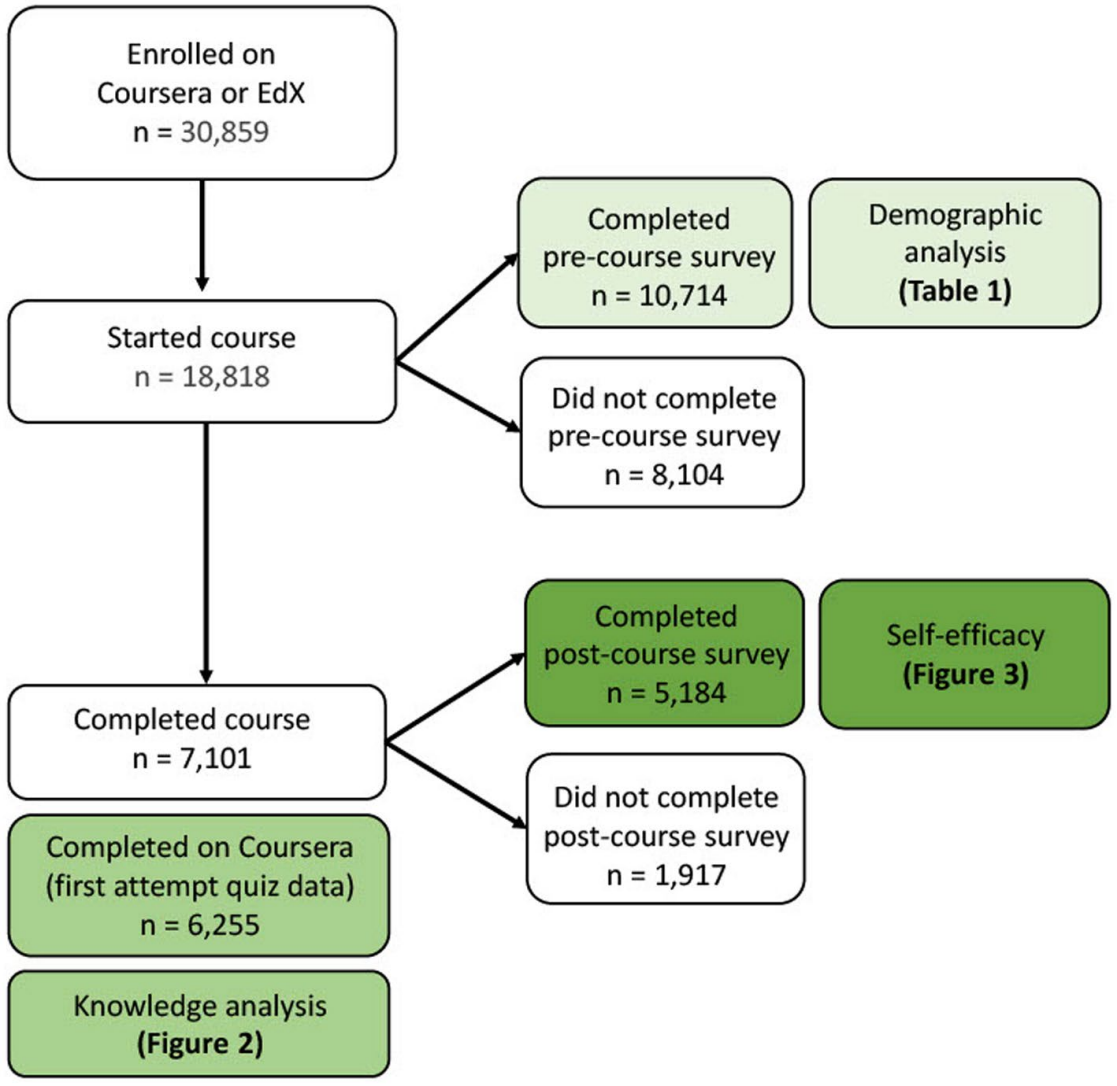
Participant enrollment and completion.

Between July 17th and September 24th, 2020, 30,859 learners enrolled in the course with 18,818 starting the course. Of those who started the course, 10,714 participated in the pre-course survey, 7,101 completed the course, and 5,184 completed the post-course survey (Figure 1). Female participants accounted for 55% of enrollees that started the course (Table 1). The majority of participants (69%) were less than 40 years of age. The median time to course completion was 4.2 days (IQR 1.0–11.9 days).

**Table 1:** Characteristics of participants who started the course.

	*N* (%)
Age (*N* = 10,323)	
18–39 years	7,353 (71%)
40–59 years	2,445 (24%)
60 years or older	525 (5%)
Gender (*N* = 10,587)	
Male	4,325 (41%)
Female	6,161 (58%)
Other	101 (1%)
Profession (*N* = 10,264)	
Healthcare (non-physician/nurse)	3,346 (33%)
Physician	2,204 (21%)
Nurse	1,286 (13%)
Student (clinical)*	1,185 (12%)
Student (non-clinical)*	1,368 (13%)
Non-healthcare	875 (9%)
Context of employment (*N* = 10,185)	
Hospital/Inpatient	3,891 (38%)
Non-hospital/Outpatient	3,428 (34%)
Non-healthcare	2,866 (28%)
Highest level of education (*N* = 9,768)	
High school degree or less	1,982 (20%)
College degree	3,060 (31%)
Master’s degree	1,419 (15%)
Doctorate, professional, or medical degree	3,307 (34%)
Race (*N* = 9,838)	
Arab	600 (6%)
Black, African, or African American	1,041 (11%)
East Asian	557 (6%)
Hispanic	1,848 (19%)
South or Southeast Asian	3,148 (32%)
White or Caucasian	1,855 (19%)
Other	789 (8%)
World Bank Income Group (*N* = 10,662)	
High income	4,299 (40%)
Upper middle income	2,183 (20%)
Lower middle income	4,041 (38%)
Low income	139 (1%)
Geographic Region (*N* = 10,662)	
East Asia & Pacific	1,130 (11%)
Europe & Central Asia	957 (9%)
Latin America & Caribbean	1,920 (18%)
Middle East & North Africa	855 (8%)
North America	2,638 (25%)
South Asia	2,489 (23%)
Sub-Saharan Africa	673 (6%)

^*^Students were categorized as clinical if they reported working in an inpatient or outpatient clinical context. Due to rounding, percentages may not sum to 100.

### Participants by provider type

MOOC participants were predominantly healthcare workers and held higher education degrees. Healthcare workers that were neither physicians nor nurses accounted for the highest percentage of course participants who started the course (33%) while physicians accounted for one-fifth of all learners (Table 1). Students made up one quarter of participants. This included both those who identified as working at a clinical site and those that reported not practicing clinically. Non-healthcare workers accounted for 9%.

### Geographic distribution of participants

A plurality of participants (40%) came from high-income countries (HICs) followed closely by lower-middle (38%) and upper-middle-income (20%) countries (Table 1). Participation by geographic region was highest in North America (25%) but closely followed by South Asia (24%) and Latin America and the Caribbean (17%). The smallest percentage of participants came from Sub-Saharan Africa (6%).

### Knowledge gain

**Figure 2:**
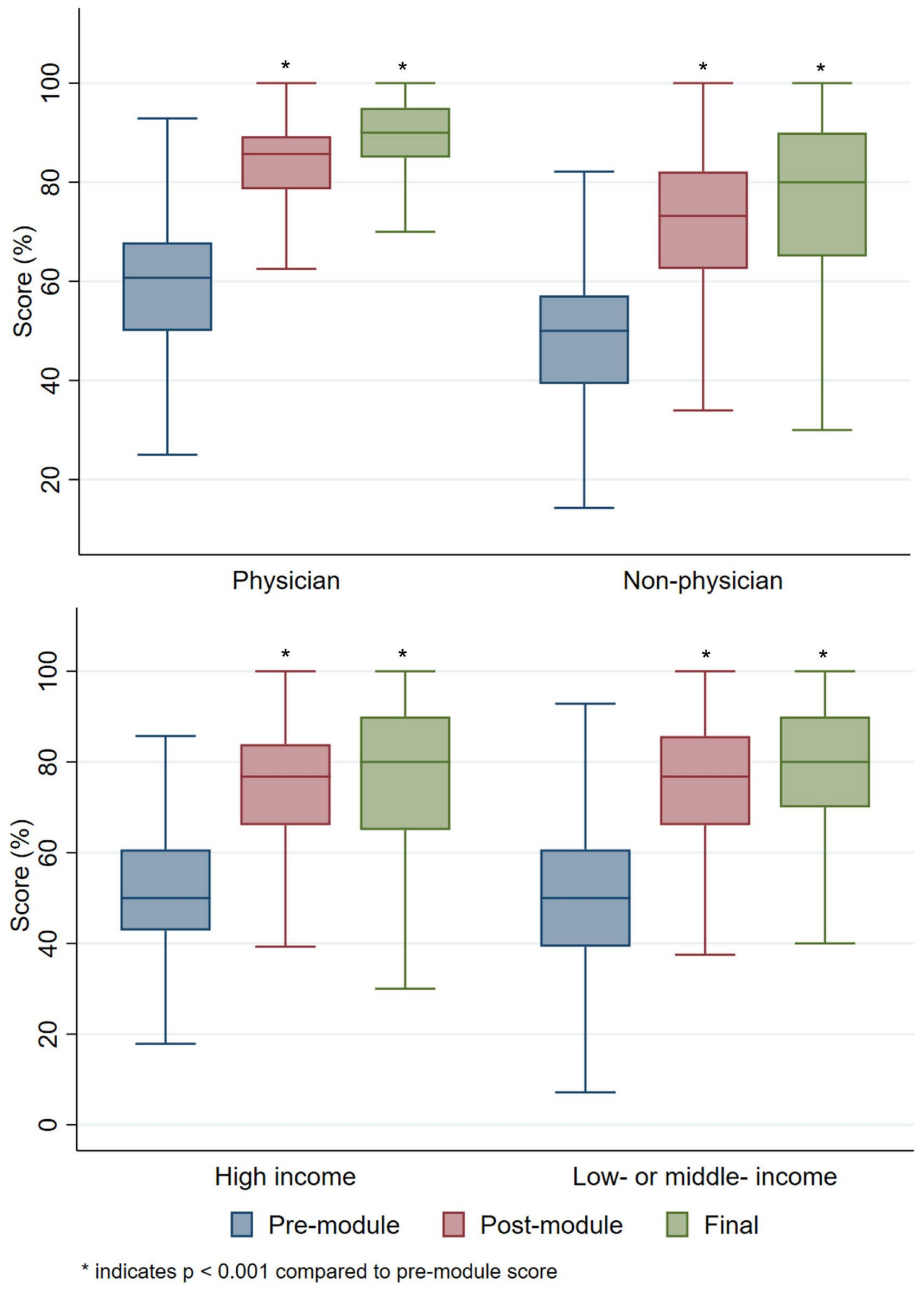
Knowledge gain by provider type and country income level.

Overall course participants demonstrated significant improvement in knowledge upon course completion. While the mean score of pre-module quizzes was 52%, participants averaged 74% on post-module quizzes and 78% on the final exam (mean difference pre vs. post 23% [*p* < 0.001] and pre vs. final 26% [*p* < 0.001]). Non-physician healthcare workers and students demonstrated knowledge gain on par with physician participants (Figure 2). Similar improvements in knowledge were obtained by participants from HICs compared to LMICs, with participants in LMICs demonstrating slightly greater improvements in knowledge (mean difference, pre vs. post: 23% LMICs vs. 22% HICs (*p* < 0.001) and pre vs. final: 27% LMICs vs. 25% HICs [*p* < 0.001]) (Figure 2).

### Predictors of course completion

On multivariable analysis, participants from low-middle and high-middle-income countries were more likely to complete the course compared to learners from HICs (Table 2). Physicians, nurses and students had similar course completion rates with lower completion rates noted in other healthcare workers and non-healthcare workers. Additionally, participants reporting female gender and from 40 to 59 years old, compared with those <39 years of age, were less likely to complete the course.

**Table 2:** Course completion rates by characteristic^a^.

Characteristic	Adjusted odds ratio	Std. Err.	*p*-value	[95% Conf. interval]
Age				
18–39 years	Ref			
40–59 years	0.894	0.049	0.042	0.802–0.996
60 years or older	1.009	0.105	0.929	0.824–1.237
Gender				
Male	Ref			
Female	0.869	0.039	0.002	0.795–0.95
Other	0.764	0.179	0.251	0.482–1.21
Profession				
Physician	Ref			
Nurse	1.158	0.100	0.090	0.977–1.371
Other healthcare (non-physician/nurse)	0.835	0.057	0.008	0.731–0.954
Student (clinical)	1.159	0.101	0.090	0.977–1.374
Student (non-clinical)	0.877	0.101	0.254	0.700–1.099
Non-healthcare	0.602	0.067	<0.001	0.484–0.748
Context of employment				
Hospital/inpatient	Ref			
Non-hospital/outpatient	0.924	0.048	0.125	0.835–1.022
Non-healthcare	0.904	0.073	0.214	0.771–1.060
Highest education level				
Doctorate, professional, or medical degree	Ref			
Master’s degree	0.830	0.061	0.011	0.719–0.959
College degree	0.946	0.057	0.357	0.840–1.065
High school degree or less	1.063	0.075	0.385	0.926–1.221
World Bank Income Group				
High income	Ref			
Upper middle income	1.087	0.066	0.171	0.965–1.226
Lower middle income	1.177	0.062	0.002	1.062–1.304
Low income	0.739	0.144	0.120	0.505–1.082
*N*	8,945			
Likelihood ratio chi-squared (G^2^)	159.32			
Pseudo *R*^2^	0.013			

^a^Multivariate logistic regression, univariate regression model provided in Supplementary Appendices.

### Survey results

**Figure 3:**
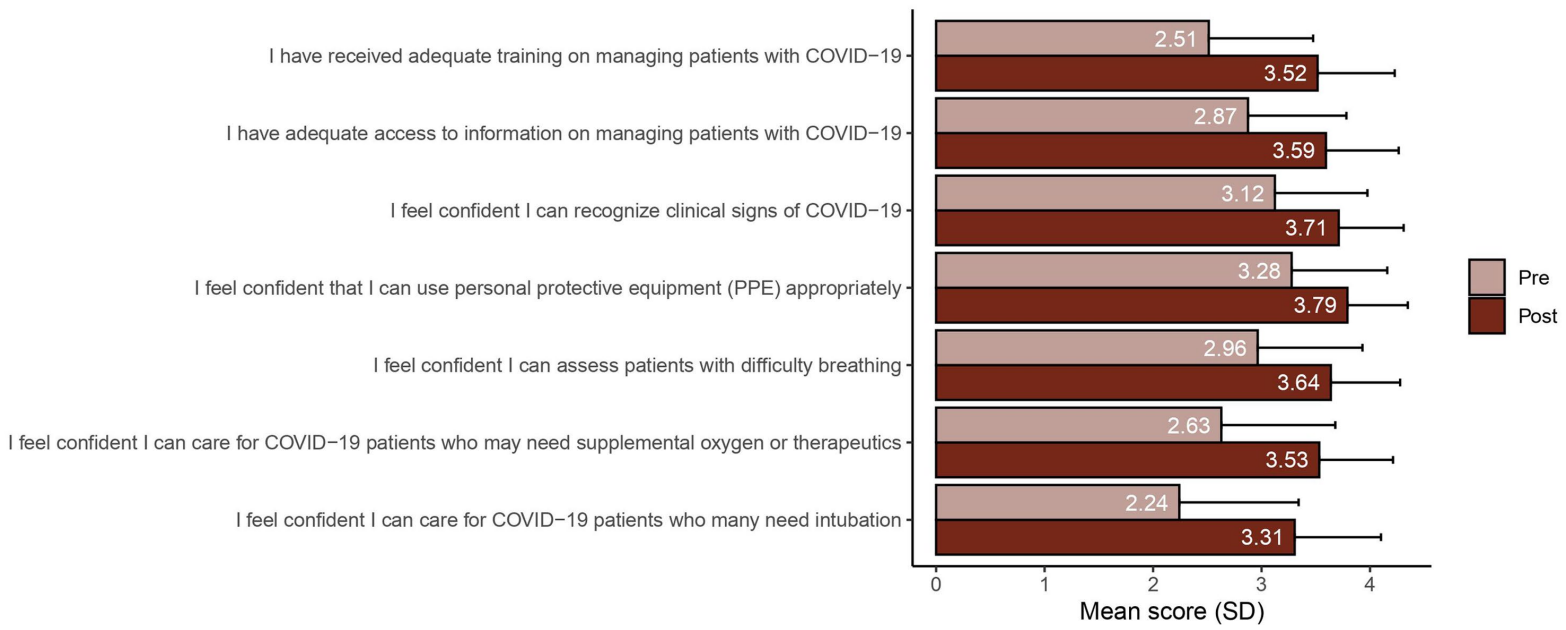
Learner self-assessment of training and access to information.

Following course completion, there were substantial improvements in learner confidence in caring for COVID-19 patients and in their self-assessment of both the adequacy of their training and access to information regarding COVID-19 (Figure 3). Further, healthcare workers who completed the course strongly agreed that the course was relevant and provided them with new knowledge about COVID-19. Most learners (92.5%) stated they were likely to recommend this course to their colleagues.

## Discussion

### Principle findings

This article demonstrates that MOOCs can effectively reach practicing healthcare workers in both high-income and middle-income countries and provide timely clinical training during a healthcare crisis. Providers from a multitude of backgrounds sought out self-directed clinical training on the recognition and care of COVID-19 patients, with a high percentage of learners completing the course. Survey findings revealed that knowledge scores improved regardless of provider background and geography. Supplementing these gains, providers reported increased confidence in their clinical skills to care for COVID-19 patients as well as the availability of both relevant and accurate information regarding the pandemic.

### Findings in context

Just-in-time learning during a pandemic or other health crisis is imperative to improving patient outcomes and protecting the healthcare workforce. While healthcare resources have been strained in many environments during the pandemic, those in LMICs and rural and remote areas are scarce at baseline, making their response to the pandemic even more challenging (27–32). Furthermore, HCWs in LMICs may experience lower access to pandemic-related training, potentially impacting preparedness and quality of care (33). The immediate necessity of HCW training during the pandemic adds substantially to the ongoing burden of delivering quality care to patients. However, with the rapid rise in access to smartphones over the past decade, mobile online training has become feasible and accepted in many settings (34). This approach allows for the development of rapidly scalable training programs with the potential for broad distribution, without taxing the already-limited local resources.

Evans and colleagues reported on a MOOC deployed during the Ebola outbreak in 2014–2015 aimed at educating the general population on the virus. One-third of their learners came from developing economies, however, only a small fraction of their learners came from nations with active Ebola outbreaks and they did not report specifically on HCW participation or knowledge gain (35). Sneddon et al. (20) conducted a MOOC aimed at improving HCW antimicrobial stewardship. While 70% of their learners were HCWs, the course was administered as live participation over 6 weeks and assessment of knowledge gain and course efficacy by participant clinical job, demographic factors, or country income level were not evaluated.

During the COVID-19 pandemic in addition to our course, other international health organizations, universities, and nations have launched online training programs that have enrolled large numbers of users (5, 36, 37). Utenen et al. launched a course on the OpenWHO platform that covered multiple topics related to COVID-19, from emerging respiratory viruses to community engagement (5). This MOOC experienced tremendous enrollment in both the Spanish and English versions of the course. Interestingly, the Spanish version of the course had a dramatically higher completion rate (36%) than the English version (3%). Sixty percent of users were listed as “Other” or “Student” and they did not specifically report the percent of HCWs practicing clinically nor include knowledge assessments. The distribution of users accessing the OpenWHO COVID-19 course, our offering, and other similar MOOCs has repeatedly skewed toward middle- and high-income-countries. While governments in low-income countries (LICs) have implemented pandemic-related information dissemination and contact tracing through mobile applications, pandemic-related online education for HCW in LICs remains relatively underexplored (38). While MOOC’s can clearly reach healthcare workers in these nations, it remains unknown whether open-access online training solutions are equally viable options in low-income countries (LICs), given limited data due to low enrollment of providers from LICs.

Encouragingly, course completion rates, knowledge scores, and survey responses all suggested — despite the course materials being developed in English and mostly by providers based in the United States — that the course was effective across diverse geographic locals. In contrast to the typical <10% completion rates among MOOCs, our course had a completion rate of 38% (39). Additionally, knowledge scores improved significantly regardless of provider geography. The improvement in knowledge was also reflected in learner sentiment, with over 90% of learners reporting the receipt of adequate training and improved confidence in their ability to care for patients with COVID-19. These positive findings were consistent for non-physician healthcare workers too, as this cohort reported strong improvements in knowledge and self-efficacy.

Non-physician providers make up the majority of all healthcare workers, and account for even higher percentages of the workforce in LMICs (40). A plurality (31%) of learners in our program were healthcare workers representing neither physicians (21%) nor nurses (12%), such as pharmacists (3%) and emergency medical technicians (2%). The diverse training backgrounds of our enrolled providers, including non-physician healthcare workers, attests to their substantial interest in clinical training during a global health crisis; and, a free-of-charge MOOC effectively contributed to meeting this widespread demand.

Several factors may have contributed to the course’s relatively high completion rate, knowledge gain, and feedback scores documented in this report. First, our education and design teams have extensive experience in delivering both in-person and online content to healthcare providers from across the globe. This experience enabled course creators to adopt a versatile style that incorporated limited text, simple language, references to trusted materials, and highly focused content with direct relevance to clinical practice. Second, unlike traditional MOOCs, which often take months to complete, our MOOC was developed with an eye toward practicing healthcare workers whose time for continuing education was extremely limited and who required information on COVID-19 immediately. In line with self-directed learning theory, the entire course was designed to be completed within 5 hours, and its duration was segmented into brief modules requiring <15 min of continuous engagement. This time-sensitive format was intended to boost engagement from providers in LMICs, who might have restricted availability (due to job or home commitments) or unreliable internet access. Third, we were able to offer the course and certification free of charge (Coursera) or at an extremely reduced cost (EdX). Many non-physician healthcare workers in LMICs are often living near the poverty line themselves and cost may heavily influence their ability to access training programs.

### Challenges and next steps

Our evaluation found the course was successful on many fronts, however, a number of limitations exist. First, the COVID-19 pandemic has brought with it innumerable challenges, one of which is the deluge of information, and misinformation, available to individuals and communities (22–24). The rapidly evolving evidence used to guide best clinical practices along with the disparate recommendations from local, national, and international health organizations, particularly early on during the pandemic, made it challenging to produce educational materials that could gain broad acceptance yet contain clinically actionable information. Furthermore, as information evolves, keeping materials and guidelines up to date poses a significant challenge, particularly at an international level (41). Even when course materials are kept current, providers that have completed the course may not continue to engage with new or revised materials, leading to knowledge decay (42).

Second, while our findings of short-term knowledge gain and improvements in participant self-assessment are encouraging, they may not be clinically meaningful. Future research is required to evaluate if inservice training for healthcare workers via a MOOC during a healthcare crisis improves long term knowledge, clinical decision making and patient-oriented outcomes.

Third, while thousands of healthcare workers were trained, millions more healthcare workers are practicing on the frontlines and require clinically relevant information during the pandemic. From our results, it is unclear if MOOCs can effectively reach providers in low-income countries and in rural and remote environments, where access to the internet may be tenuous or nonexistent. Alternative course delivery options and additional languages are being launched as prior evidence demonstrates that offering MOOCs in multiple languages can dramatically increase enrollment (5).

## Conclusion

Future pandemics and outbreaks will require a rapid, consolidated global response to minimize patient morbidity and mortality. Several strategies have been identified to prevent further escalation to the level of a global health crisis, notably HCW education on infection prevention and control strategies (43). Given the country-to-country variability regarding infection prevention guidelines, MOOCS can address the need for rapid, standardized access to emerging medical knowledge during public health crises (41).

This study demonstrates that MOOCs can effectively reach practicing HCWs in low-income countries and provide inservice training during a global health crisis. Physician and non-physician providers from a multitude of geographies and backgrounds sought out self-directed clinical training on recognizing and caring for COVID-19 patients with a high percentage of learners completing the course. Knowledge improved across all participant groups regardless of demographic and other characteristics. Future research is required to understand the impact on patient-oriented outcomes and how to better reach HCWs in low-income countries.

## Data Availability

The raw data supporting the conclusions of this article will be made available by the authors, without undue reservation.
