# Supplementary figures and images for: Evaluation of a massive open online course for just-in-time training of healthcare workers
Source: Front Public Health. 2024 Oct 1;12:1395931. doi: 10.3389/fpubh.2024.1395931 (PMC11478164; doi:10.3389/fpubh.2024.1395931)

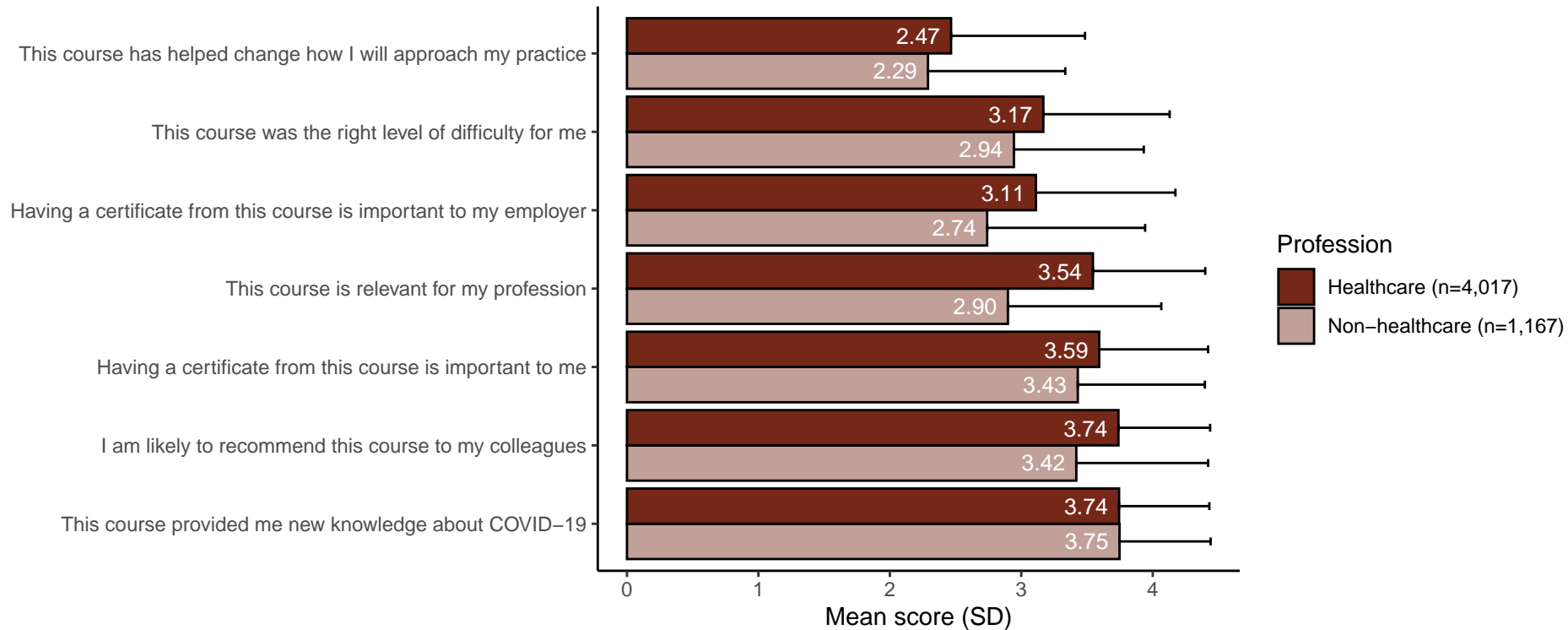

Supplement: Supplementary file 1 [file Data_Sheet_1.PDF]
