# Supplementary material for: Evaluation of a massive open online course for just-in-time training of healthcare workers
Source: Front Public Health. 2024 Oct 1;12:1395931. doi: 10.3389/fpubh.2024.1395931 (PMC11478164; doi:10.3389/fpubh.2024.1395931)
Supplement: Supplementary file 2 [file Table_1.DOCX]

**Appendix 1: Course Surveys**

**PRE-COURSE SURVEY**

**Complete your learner profile.**

Before you begin the course, we would love to learn more about you. This will help us better understand who is taking the course and how to improve the course to better serve our learners.

Your participation is voluntary. You can choose not to answer any question, and your responses will not affect your performance in the course and the certification process. The information you share with us is completely confidential and your risks for responding are minimal. We may use the information we collect as part of this survey in program materials or in research reports; however, none of the information you share will be associated with your identity. All information collected will be reported in combination with other responses so that no individual can be identified.

Would you like to complete the learner profile survey?

1. Yes - If yes, please proceed below.
2. No - If no, please skip to the next section of the course.
3. What best describes your profession? Select all that apply.
   1. Community Health Worker
   2. Emergency Medical Technician (EMT)
   3. Nurse
   4. Pharmacist
   5. Physician
   6. Physician assistant
   7. Physical or occupational therapist
   8. Social worker
   9. Student
   10. Other healthcare professional
   11. Other non healthcare professional
4. In which of the following contexts do you work? Select all that apply.
   1. Hospital
   2. Emergency department / casualty ward
   3. Outpatient clinic
   4. Patient homes
   5. Skilled nursing facility
   6. Telemedicine
   7. Other healthcare setting
   8. None of the above
5. Do you currently work with COVID-19 patients?
   1. Yes
   2. No
6. Do you anticipate that you will work with COVID-19 patients in the future?
   1. Yes
   2. No
7. Have you been infected with COVID-19?
   1. Yes
   2. No
   3. Prefer not to answer
8. In 2-3 sentences, why are you interested in taking this course? [open text]
9. In 2-3 sentences, what concerns you most about working with COVID-19 patients? [open text]
10. Have you experienced any of the following in your healthcare setting: Select all that apply.
    1. Lack of health workers
    2. Lack of PPE for health workers
    3. Lack of hospital beds and medical equipment for sick patients
    4. Lack of training for health workers
    5. None of the above
    6. Not applicable
11. Which of the following have you observed in the communities where you work? Select all that apply.
    1. Lack of public information about COVID-19 for low literacy populations
    2. Lack of public information about COVID-19 in local languages
    3. Lack of resources (such as running water) to comply with prevention guidelines
    4. Barriers to social distancing (such as close living quarters)
    5. False information and myths about COVID-19
    6. Ambivalence or a false sense of security about COVID-19
    7. Politically motivated resistance to following COVID-19 recommendations (such as wearing of masks)
    8. None of the above
12. What are your biggest concerns about the effects of COVID-19 in the communities where you work? Select all that apply
    1. Delaying of treatment for other medical issues
    2. Delaying of vaccinations
    3. Economic hardship
    4. Food insecurity
    5. Increased domestic violence
    6. Increased alcohol consumption
    7. Mental health declines
    8. Direct effects of COVID-19 illness
13. Do you have any other concerns you have about COVID-19 in the communities where you work? [open text]

How strongly do you agree with the following statements?

1. I have received adequate training on managing patients with COVID-19.
2. I have adequate access to information on managing patients with COVID-19.
3. I feel confident I can recognize clinical signs of COVID-19.
4. I feel confident I can assess patients with difficulty breathing.
5. I feel confident I can care for COVID-19 patients who may need supplemental oxygen or therapeutics.
6. I feel confident I can care for COVID-19 patients who may need intubation or ventilation.
7. I feel confident that I can use personal protective equipment appropriately.

Options (list separately for each of the above):

- Strongly disagree
- Somewhat disagree
- Somewhat agree
- Strongly agree

1. How old are you?
   1. 18-29 years
   2. 30-39 years
   3. 40-49 years
   4. 50-59 years
   5. 60-69 years
   6. 70 years or older
   7. Prefer not to answer
2. What is the highest level of school you have completed or the highest degree you have received?
   1. No formal schooling completed
   2. Some primary or elementary school
   3. Some high school or secondary (but no degree)
   4. High school or secondary degree
   5. Some college but no degree
   6. Associate degree including occupational/technical/vocational degree
   7. Bachelor's degree (e.g., BA, AB, BS)
   8. Master's degree (e.g., MA, MS, MSW, MBA)
   9. Professional school or medical degree (e.g., MD, MBBS, RN, LPN)
   10. Doctorate degree (e.g., PhD, EdD)
3. What best describes your gender?
   1. Male
   2. Female
   3. Non-binary / third gender
   4. Prefer to self describe
   5. Other
   6. Prefer not to answer
4. What is your race or ethnicity? Select all that apply.
   1. Arab
   2. Black, African, or African American
   3. East Asian
   4. Hispanic, Latinx or Spanish Origin
   5. Native American, Native Alaskan or Indigenous
   6. Native Hawaiian or Pacific Islander
   7. South Asian
   8. Southeast Asian
   9. White or Caucasian
   10. Other
   11. Prefer not to answer
5. If you live in the United States, in which zip code do you work?

Enter “N/A” if located outside the United States.

**POST-COURSE SURVEY**

**Course Feedback Survey**

Thank you for your participation! Your feedback on the course is very valuable and will help us improve the course.

Your participation in this feedback survey is voluntary. You can choose not to answer any question, and your responses will not affect your performance in the course and the certification process. The information you share with us is completely confidential and your risks for responding are minimal. We may use the information we collect as part of this survey in program materials or in research reports; however, none of the information you share will be associated with your identity. All information collected will be reported in combination with other responses so that no individual can be identified.

Would you like to participate in the feedback survey?

1. Yes
2. No

*How strongly do you agree or disagree with the following statements?*

1. This course provided new knowledge to me about COVID-19.
2. This course was the right level of difficulty for me.
3. This course has helped change how I will approach my practice.
4. This course is relevant to my professional work
5. I am likely to recommend this course to my colleagues
6. Having a certificate from this course is important to me.
7. Having a certificate from this course is important to my employer.

Options (list separately for each of the above):

- Strongly disagree
- Somewhat disagree
- Somewhat agree
- Strongly agree
- Prefer not to answer

Thank you for your feedback. Now that you have completed the course, we would like to ask how prepared you feel to treat COVID-19 patients.

How strongly do you agree or disagree with the following statements?

1. I have received adequate training on managing patients with COVID-19.
2. I have adequate access to information on managing patients with COVID-19.
3. I feel confident I can recognize clinical signs of COVID-19.
4. I feel confident I can assess patients with difficulty breathing.
5. I feel confident I can care for COVID-19 patients who may need supplemental oxygen or therapeutics.
6. I feel confident I can care for COVID-19 patients who many need intubation or ventilation.
7. I feel confident that I can use personal protective equipment (PPE) appropriately.

Options (list separately for each of the above):

- Strongly disagree
- Somewhat disagree
- Somewhat agree
- Strongly agree
- Prefer not to answer

**Appendix 2: Descriptive and univariate logistic regression analysis of course completion**

| \|  \| Started but did not complete \| Completed course \| Odds ratio (95% CI) \| Odds ratio (95% CI) \| \| --- \| --- \| --- \| --- \| --- \| |
| --- | --- | --- | --- | --- | --- |
| \| **Age (N=10,323)** \|  \|  \|  \|  \|  \| \| --- \| --- \| --- \| --- \| --- \| --- \| \| 18-39 years \| 3676 (66%) \| 3677 (71%) \| Ref \|  \| 3213 (73%) \| \| 40-59 years \| 1365 (25%) \| 1080 (21%) \| 0.791 (0.721-0.867) \|  \| 961 (22%) \| \| 60 years or older \| 281 (5%) \| 244 (5%) \| 0.868 (0.727-1.037) \|  \| 210 (5%) \| \| **Gender** **(N=10,587)** \|  \|  \|  \|  \|  \| \| Male \| 2173 (39%) \| 2152 (42%) \| Ref \|  \| 1833 (42%) \| \| Female \| 3241 (59%) \| 2920 (56%) \| 0.91 (0.842-0.983) \|  \| 2518 (57%) \| \| Other \| 54 (1%) \| 47 (1%) \| 0.879 (0.592-1.305) \|  \| 33 (1%) \| \| **Profession (N=10,264)** \|  \|  \|  \|  \|  \| \| Physician \| 1073 (19%) \| 1131 (22%) \| Ref \|  \| 1392 (32%) \| \| Nurse \| 604 (11%) \| 682 (13%) \| 1.071 (0.933-1.229) \|  \|  \| \| Other healthcare \| 1819 (33%) \| 1527 (29%) \| 0.796 (0.715-0.887) \|  \| 957 (22%) \| \| Student (healthcare) \| 508 (9%) \| 677 (13%) \| 1.264 (1.097-1.457) \|  \| 617 (14%) \| \| Student (other) \| 698 (13%) \| 670 (13%) \| 0.911 (0.796-1.042) \|  \| 510 (12%) \| \| Non-healthcare \| 560 (10%) \| 315 (6%) \| 0.534 (0.454-0.627) \|  \| 291 (7%) \| \| **Context of employment (N=10,185)** \|  \|  \|  \|  \|  \| \| Hospital / Inpatient \| 1877 (34%) \| 2014 (39%) \| Ref \|  \| 1795 (41%) \| \| Non-hospital / Outpatient \| 1780 (32%) \| 1648 (32%) \| 0.863 (0.787-0.946) \|  \| 1519 (35%) \| \| Non-healthcare \| 1606 (29%) \| 1260 (24%) \| 0.731 (0.664-0.806) \|  \| 1070 (24%) \| \| **Highest level of education (N=9,768)** \|  \|  \|  \|  \|  \| \| Doctorate, professional, medical degree \| 1595 (29%) \| 1712 (33%) \| Ref \|  \|  \| \| Master's degree \| 822 (15%) \| 597 (12%) \| 0.677 (0.597-0.767) \|  \|  \| \| College degree \| 1624 (29%) \| 1436 (28%) \| 0.824 (0.747-0.909) \|  \| 1321 (30%) \| \| High school degree or less \| 977 (18%) \| 1005 (19%) \| 0.958 (0.857-1.071) \|  \| 545 (12%) \| \| **Race**^1^ **(N=9,768)** \|  \|  \|  \|  \|  \| \| White or Caucasian \| 1076 (19%) \| 779 (15%) \| Ref \|  \|  \| \| Arab \| 339 (6%) \| 261 (5%) \| 1.063 (0.883-1.281) \|  \| 227 (5%) \| \| Black, African, or African American \| 559 (10%) \| 482 (9%) \| 1.191 (1.022-1.388) \|  \| 435 (10%) \| \| East Asian \| 289 (5%) \| 268 (5%) \| 1.281 (1.059-1.549) \|  \| 241 (5%) \| \| Hispanic \| 907 (16%) \| 941 (18%) \| 1.433 (1.259-1.631) \|  \| 805 (18%) \| \| South or Southeast Asian \| 1481 (27%) \| 1667 (32%) \| 1.555 (1.385-1.746) \|  \| 1447 (33%) \| \| Other \| 426 (8%) \| 363 (7%) \| 1.177 (0.995-1.392) \|  \| 301 (7%) \| \| **World Bank Income Group (N=10,662)** \|  \|  \|  \|  \|  \| \| High income \| 2335 (43%) \| 1964 (38%) \| Ref \|  \| 1550 (37%) \| \| Upper middle income \| 1108 (20%) \| 1075 (21%) \| 1.153 (1.04-1.279) \|  \|  \| \| Lower middle income \| 1966 (36%) \| 2075 (40%) \| 1.255 (1.151-1.368) \|  \| 1621 (39%) \| \| Low income \| 85 (2%) \| 54 (1%) \| 0.755 (0.534-1.068) \|  \| 914 (22%) \| \| **Geographic Region (N=10,662)** \|  \|  \|  \|  \|  \| \| North America \| 1569 (29%) \| 1069 (21%) \| Ref \|  \|  \| \| East Asia & Pacific \| 543 (10%) \| 587 (11%) \| 1.587 (1.379-1.825) \|  \| 448 (11%) \| \| Europe & Central Asia \| 500 (9%) \| 457 (9%) \| 1.342 (1.156-1.557) \|  \| 354 (9%) \| \| Latin America & Caribbean \| 912 (17%) \| 1008 (20%) \| 1.622 (1.441-1.826) \|  \| 798 (19%) \| \| Middle East & North Africa \| 402 (7%) \| 453 (9%) \| 1.654 (1.416-1.932) \|  \| 380 (9%) \| \| South Asia \| 1194 (22%) \| 1295 (25%) \| 1.592 (1.425-1.778) \|  \| 1053 (25%) \| \| Sub-Saharan Africa \| 374 (7%) \| 299 (6%) \| 1.173 (0.989-1.392) \|  \| 268 (6%) \| |

^1^White or Caucasian race was used as the reference group because they have been shown to have higher completion rates

**Appendix 3: Self efficacy by profession and World Bank income group**

Mean (SD) of pre- and post-course self-efficacy scores by profession

| \|  \| Other healthcare \| Physician \| Nurse \| Student (healthcare) \| Student (other) \| Non-healthcare \| Unknown \| Total \| \| --- \| --- \| --- \| --- \| --- \| --- \| --- \| --- \| --- \| \|  \| (N = 1457) \| (N = 1091) \| (N = 676) \| (N = 668) \| (N = 672) \| (N = 293) \| (N = 174) \| (N = 5031)* \| |
| --- | --- | --- | --- | --- | --- | --- | --- | --- | --- | --- | --- | --- | --- | --- | --- | --- | --- | --- |
| \| **I have received adequate training on managing patients with COVID-19** \| \| \| \| \| \| \| \| \|  \|  \| \| --- \| --- \| --- \| --- \| --- \| --- \| --- \| --- \| --- \| --- \| --- \| \| Pre \| 2.59 (0.94) \| 2.44 (0.91) \| 2.66 (0.97) \| 2.29 (0.97) \| 2.26 (1.02) \| 2.39 (1.02) \| 2.65 (1.09) \| 2.48 (0.97) \| \|  \| \| Post \| 3.55 (0.70) \| 3.55 (0.65) \| 3.52 (0.79) \| 3.51 (0.68) \| 3.48 (0.71) \| 3.32 (0.86) \| 3.46 (0.73) \| 3.52 (0.71) \| \|  \| \| **I have adequate access to information on managing patients with COVID-19** \| \| \| \| \| \| \| \|  \| \|  \| \| Pre \| 2.82 (0.91) \| 2.95 (0.86) \| 2.91 (0.89) \| 2.76 (0.89) \| 2.66 (0.94) \| 2.55 (1.04) \| 2.62 (1.10) \| 2.82 (0.92) \| \|  \| \| Post \| 3.58 (0.70) \| 3.65 (0.59) \| 3.59 (0.73) \| 3.66 (0.60) \| 3.55 (0.66) \| 3.39 (0.80) \| 3.56 (0.69) \| 3.59 (0.67) \| \|  \| \| **I feel confident I can recognize clinical signs of COVID-19** \| \| \| \| \| \| \| \|  \| \|  \| \| Pre \| 3.04 (0.89) \| 3.18 (0.81) \| 3.14 (0.84) \| 3.05 (0.85) \| 2.94 (0.88) \| 2.74 (0.96) \| 2.70 (0.98) \| 3.05 (0.87) \| \|  \| \| Post \| 3.69 (0.64) \| 3.83 (0.47) \| 3.71 (0.65) \| 3.72 (0.58) \| 3.65 (0.57) \| 3.53 (0.68) \| 3.62 (0.67) \| 3.71 (0.60) \| \|  \| \| **I feel confident that I can use personal protective equipment (PPE) appropriately** \| \| \| \| \| \| \| \|  \| \|  \| \| Pre \| 3.27 (0.90) \| 3.18 (0.80) \| 3.44 (0.81) \| 3.11 (0.90) \| 2.98 (1.02) \| 3.13 (0.98) \| 3.08 (0.87) \| 3.21 (0.89) \| \|  \| \| Post \| 3.78 (0.59) \| 3.86 (0.43) \| 3.79 (0.62) \| 3.80 (0.54) \| 3.74 (0.55) \| 3.71 (0.64) \| 3.75 (0.60) \| 3.79 (0.56) \| \|  \| \| **I feel confident I can assess patients with difficulty breathing** \| \| \| \| \| \| \| \|  \| \|  \| \| Pre \| 2.91 (0.98) \| 3.10 (0.87) \| 3.24 (0.90) \| 2.67 (0.95) \| 2.58 (0.99) \| 2.53 (1.08) \| 2.86 (0.98) \| 2.91 (0.97) \| \|  \| \| Post \| 3.60 (0.67) \| 3.80 (0.48) \| 3.67 (0.69) \| 3.62 (0.62) \| 3.55 (0.62) \| 3.37 (0.76) \| 3.54 (0.73) \| 3.64 (0.64) \| \|  \| \| **I feel confident I can care for COVID-19 patients who may need supplemental oxygen or therapeutics** \| \| \| \| \| \| \| \|  \| \|  \| \| Pre \| 2.57 (1.07) \| 2.71 (0.94) \| 3.05 (0.96) \| 2.37 (1.00) \| 2.29 (1.04) \| 2.12 (1.07) \| 2.75 (1.15) \| 2.59 (1.04) \| \|  \| \| Post \| 3.50 (0.70) \| 3.70 (0.55) \| 3.61 (0.70) \| 3.50 (0.65) \| 3.44 (0.67) \| 3.17 (0.85) \| 3.38 (0.81) \| 3.53 (0.68) \| \|  \| \| **I feel confident I can care for COVID-19 patients who many need intubation or ventilation** \| \| \| \| \| \| \| \|  \| \|  \| \| Pre \| 2.23 (1.11) \| 2.24 (1.02) \| 2.60 (1.11) \| 2.06 (1.04) \| 2.10 (1.09) \| 1.89 (1.09) \| 2.47 (1.15) \| 2.23 (1.09) \| \|  \| \| Post \| 3.27 (0.81) \| 3.39 (0.72) \| 3.39 (0.81) \| 3.32 (0.73) \| 3.29 (0.76) \| 2.89 (0.98) \| 3.18 (0.97) \| 3.31 (0.80) \| \|  \| |

* includes only those who completed both pre and post-course self-efficacy questions

Mean (SD) of pre- and post-course self-efficacy scores by World Bank Income Group

| \|  \| High \| Upper middle \| Lower middle \| Low \| Unknown \| Total \| \| --- \| --- \| --- \| --- \| --- \| --- \| --- \| \|  \| (N = 1908) \| (N = 1045) \| (N = 2014) \| (N = 48) \| (N = 16) \| (N = 5031)* \| |
| --- | --- | --- | --- | --- | --- | --- | --- | --- | --- | --- | --- | --- | --- | --- |
| \| **I have received adequate training on managing patients with COVID-19** \| \| \| \| \| \|  \| \| --- \| --- \| --- \| --- \| --- \| --- \| --- \| \| Pre \| 2.50 (0.97) \| 2.45 (0.96) \| 2.48 (0.97) \| 2.41 (0.93) \| 2.00 (1.15) \| 2.48 (0.97) \| \| Post \| 3.45 (0.75) \| 3.59 (0.69) \| 3.54 (0.68) \| 3.62 (0.65) \| 2.78 (1.09) \| 3.52 (0.71) \| \| **I have adequate access to information on managing patients with COVID-19** \| \| \| \| \| \|  \| \| Pre \| 2.83 (0.91) \| 2.93 (0.92) \| 2.76 (0.91) \| 2.87 (0.91) \| 2.33 (0.98) \| 2.82 (0.92) \| \| Post \| 3.55 (0.71) \| 3.68 (0.61) \| 3.59 (0.66) \| 3.48 (0.71) \| 3.67 (0.50) \| 3.59 (0.67) \| \| **I feel confident I can recognize clinical signs of COVID-19** \| \| \| \| \| \|  \| \| Pre \| 3.01 (0.87) \| 3.09 (0.86) \| 3.07 (0.88) \| 3.15 (0.87) \| 2.29 (0.83) \| 3.05 (0.87) \| \| Post \| 3.68 (0.63) \| 3.76 (0.57) \| 3.72 (0.58) \| 3.83 (0.57) \| 3.78 (0.44) \| 3.71 (0.60) \| \| **I feel confident that I can use personal protective equipment (PPE) appropriately** \| \| \| \| \| \|  \| \| Pre \| 3.32 (0.84) \| 3.16 (0.93) \| 3.13 (0.91) \| 3.38 (0.90) \| 2.67 (0.49) \| 3.21 (0.89) \| \| Post \| 3.78 (0.58) \| 3.81 (0.56) \| 3.79 (0.54) \| 3.89 (0.53) \| 3.89 (0.33) \| 3.79 (0.56) \| \| **I feel confident I can assess patients with difficulty breathing (Pre)** \| \| \| \| \| \|  \| \| Pre \| 2.98 (0.96) \| 2.76 (1.01) \| 2.92 (0.95) \| 3.00 (1.05) \| 2.42 (0.79) \| 2.91 (0.97) \| \| Post \| 3.60 (0.66) \| 3.66 (0.64) \| 3.67 (0.61) \| 3.63 (0.60) \| 3.67 (0.71) \| 3.64 (0.64) \| \| **I feel confident I can care for COVID-19 patients who may need supplemental oxygen or therapeutics** \| \| \| \| \| \|  \| \| Pre \| 2.62 (1.06) \| 2.51 (1.05) \| 2.62 (1.03) \| 2.67 (0.98) \| 2.00 (0.95) \| 2.59 (1.04) \| \| Post \| 3.48 (0.73) \| 3.56 (0.66) \| 3.56 (0.64) \| 3.51 (0.66) \| 3.33 (0.87) \| 3.53 (0.68) \| \| **I feel confident I can care for COVID-19 patients who many need intubation or ventilation** \| \| \| \| \| \|  \| \| Pre \| 2.23 (1.11) \| 2.13 (1.07) \| 2.29 (1.08) \| 2.29 (1.01) \| 2.11 (0.78) \| 2.23 (1.09) \| \| Post \| 3.21 (0.87) \| 3.31 (0.77) \| 3.38 (0.73) \| 3.24 (0.87) \| 3.33 (1.12) \| 3.31 (0.80) \| |

* includes only those who completed both pre and post-course self efficacy questions

**Appendix 4: Course feedback by profession and World Bank income group**

Mean (SD) post-course feedback scores, by profession

| \|  \| Other healthcare \| Physician \| Nurse \| Student (clinical) \| Student (non-clinical) \| Non-healthcare \| Unknown \| Total \| \| --- \| --- \| --- \| --- \| --- \| --- \| --- \| --- \| --- \| \|  \| (N = 1527) \| (N = 1131) \| (N = 682) \| (N = 677) \| (N = 670) \| (N = 315) \| (N = 182) \| (N = 5184) \| |
| --- | --- | --- | --- | --- | --- | --- | --- | --- | --- | --- | --- | --- | --- | --- | --- | --- | --- | --- |
| \| This course provided me new knowledge about COVID-19 \| 3.82 (0.72) \| 3.73 (0.73) \| 3.80 (0.75) \| 3.82 (0.65) \| 3.76 (0.71) \| 3.91 (0.62) \| 3.72 (0.88) \| 3.79 (0.72) \| \| --- \| --- \| --- \| --- \| --- \| --- \| --- \| --- \| --- \| \|  \|  \|  \|  \|  \|  \|  \|  \|  \| \| I am likely to recommend this course to my colleagues \| 3.69 (0.83) \| 3.86 (0.55) \| 3.81 (0.70) \| 3.80 (0.63) \| 3.54 (0.92) \| 3.32 (1.12) \| 3.34 (1.18) \| 3.70 (0.80) \| \|  \|  \|  \|  \|  \|  \|  \|  \|  \| \| Having a certificate from this course is important to me \| 3.64 (0.87) \| 3.61 (0.83) \| 3.64 (0.87) \| 3.66 (0.80) \| 3.59 (0.88) \| 3.36 (1.10) \| 3.31 (1.14) \| 3.60 (0.88) \| \|  \|  \|  \|  \|  \|  \|  \|  \|  \| \| This course is relevant to my professional work \| 3.36 (1.01) \| 3.74 (0.72) \| 3.68 (0.83) \| 3.76 (0.69) \| 3.31 (1.12) \| 2.63 (1.35) \| 2.96 (1.37) \| 3.47 (0.99) \| \|  \|  \|  \|  \|  \|  \|  \|  \|  \| \| This course was the right level of difficulty for me \| 3.14 (1.04) \| 3.37 (0.95) \| 3.30 (1.04) \| 3.25 (0.99) \| 3.04 (1.05) \| 3.05 (0.99) \| 2.89 (1.19) \| 3.20 (1.02) \| \|  \|  \|  \|  \|  \|  \|  \|  \|  \| \| Having a certificate from this course is important to my employer \| 3.23 (1.13) \| 3.21 (1.16) \| 3.27 (1.11) \| 3.29 (1.11) \| 2.99 (1.28) \| 2.97 (1.37) \| 2.85 (1.45) \| 3.18 (1.18) \| \|  \|  \|  \|  \|  \|  \|  \|  \|  \| \| This course has helped change how I will approach my practice \| 2.37 (1.04) \| 2.53 (1.01) \| 2.49 (1.01) \| 2.56 (0.98) \| 2.32 (1.06) \| 2.16 (1.03) \| 2.72 (1.22) \| 2.44 (1.04) \| \|  \|  \|  \|  \|  \|  \|  \|  \|  \| |

Mean (SD) post-course feedback scores, by World Bank Income Group

| \|  \| High \| Upper Middle \| Lower middle \| Low \| Unknown \| Total \| \| --- \| --- \| --- \| --- \| --- \| --- \| --- \| \|  \| (N = 1964) \| (N = 1075) \| (N = 2075) \| (N = 54) \| (N = 16) \| (N = 5184) \| |
| --- | --- | --- | --- | --- | --- | --- | --- | --- | --- | --- | --- | --- | --- | --- |
| \| This course provided me new knowledge about COVID-19 \| 3.77 (0.78) \| 3.81 (0.68) \| 3.81 (0.68) \| 3.76 (0.71) \| 3.78 (0.44) \| 3.79 (0.72) \| \| --- \| --- \| --- \| --- \| --- \| --- \| --- \| \|  \|  \|  \|  \|  \|  \|  \| \| Having a certificate from this course is important to me \| 3.48 (0.98) \| 3.70 (0.79) \| 3.66 (0.83) \| 3.68 (0.78) \| 3.56 (0.73) \| 3.60 (0.88) \| \|  \|  \|  \|  \|  \|  \|  \| \| Having a certificate from this course is important to my employer \| 3.11 (1.24) \| 3.36 (1.13) \| 3.15 (1.15) \| 3.50 (0.95) \| 3.78 (0.67) \| 3.18 (1.18) \| \|  \|  \|  \|  \|  \|  \|  \| \| This course has helped change how I will approach my practice \| 2.34 (1.08) \| 2.56 (1.02) \| 2.45 (1.00) \| 2.76 (0.83) \| 3.78 (0.44) \| 2.44 (1.04) \| \|  \|  \|  \|  \|  \|  \|  \| \| This course was the right level of difficulty for me \| 3.19 (1.03) \| 3.28 (0.98) \| 3.16 (1.04) \| 3.13 (1.12) \| 3.78 (0.44) \| 3.20 (1.02) \| \|  \|  \|  \|  \|  \|  \|  \| \| I am likely to recommend this course to my colleagues \| 3.61 (0.90) \| 3.83 (0.63) \| 3.72 (0.78) \| 3.87 (0.47) \| 4.00 (0.00) \| 3.70 (0.80) \| \|  \|  \|  \|  \|  \|  \|  \| \| This course is relevant to my professional work \| 3.33 (1.09) \| 3.65 (0.83) \| 3.51 (0.96) \| 3.74 (0.79) \| 3.56 (0.53) \| 3.47 (0.99) \| \|  \|  \|  \|  \|  \|  \|  \| |

**Appendix 5: Knowledge scores by profession**

Mean (SD) pre-module, post-module, and final exam scores by profession

| \|  \| Healthcare \| Non-healthcare \| Total \| \| --- \| --- \| --- \| --- \| \|  \| (N = 5080) \| (N = 1175) \| (N = 6,255) \| |
| --- | --- | --- | --- | --- | --- | --- | --- | --- |
| \| **Pre-module quizzes** \|  \|  \|  \| \| --- \| --- \| --- \| --- \| \| Mean (SD) \| 0.53 (0.15) \| 0.47 (0.15) \| 0.52 (0.15) \| \| **Post-module quizzes** \|  \|  \|  \| \| Mean (SD) \| 0.76 (0.13) \| 0.70 (0.13) \| 0.74 (0.14) \| \| **Final exam score** \|  \|  \|  \| \| Mean (SD) \| 0.79 (0.17) \| 0.73 (0.18) \| 0.78 (0.17) \| \| **Pre- and post-module difference** \|  \|  \|  \| \| Mean (SD) \| 0.23 (0.13) \| 0.24 (0.14) \| 0.23 (0.13) \| \| **Pre-module and final difference** \|  \|  \|  \| \| Mean (SD) \| 0.26 (0.16) \| 0.26 (0.18) \| 0.26 (0.17) \| |
